# A comparison of the large‐scale gene expression patterns in summer and fall migratory *Pantala flavescens* (Fabricius) in northern China

**DOI:** 10.1002/ece3.70147

**Published:** 2024-08-06

**Authors:** Lingzhen Cao, Na Wang

**Affiliations:** ^1^ College of Life Science Jiangxi Normal University Nanchang Jiangxi Province China

**Keywords:** differentially expressed genes, insect migration, Odonata, transcriptome data

## Abstract

*Pantala flavescens* (Fabricius) is the most well‐known seasonal migratory insect. This research focused on the molecular response of *P. flavescens* migration in summer and fall. A total of 17,810 assembled unigenes were obtained and 624 differentially expressed genes (DEGs) were identified in summer migration compared to fall migration. A number of DEGs, including *cpr49Ae, itm2b, chitinase, cpr11B, laccase2, nd5*, *vtg*2 and so on, had previously been reported to be involved in cold‐ and high‐temperature resistance. Functional enrichment analysis showed three pathways ‘that antibacterial humoral response, response to bacterial, and lipid transporter activity’ were significantly enriched in summer migration while that six pathways ‘structural constituent of cuticle, chitin binding, mitochondrion, propanoate metabolism, citrate cycle, hypertrophic cardiomyopathy’ were significantly enriched in fall migration. These results will provide a valuable baseline for further understanding of the molecular mechanisms of insect adaptation to different climate migrations.

## Graphical abstract

*Pantala flavescens* (Fabricius) is the most well‐known seasonal migratory insect. A total of 624 differentially expressed genes (DEGs) with log_2_FC ≥ 2 were identified in summer migration compared to fall migration. Enrichment analysis showed that immunity, bacterial response and detoxification substances were significantly enriched in summer migration while cuticle protein, mitochondrion, propanoate metabolism, citrate cycle and hypertrophic cardiomyopathy were significantly enriched in fall migration.

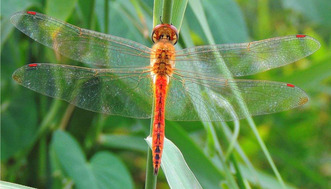

## 1 INTRODUCTION

Insects are ectotherms sensitive to temperature changes. Almost every aspect of insect physiology and behaviour can be influenced by temperature (Cui et al., 2018). Heat and cold stress change insect nervous, respiration, metabolism and endocrine systems. When insects face abiotic environmental stress, such as extreme temperature, they adopt various adaptive strategies, including diapause and migration, to escape unfavourable seasonal conditions (Chapman et al., 2015; Satterfield et al., 2020).

**FIGURE 1:**
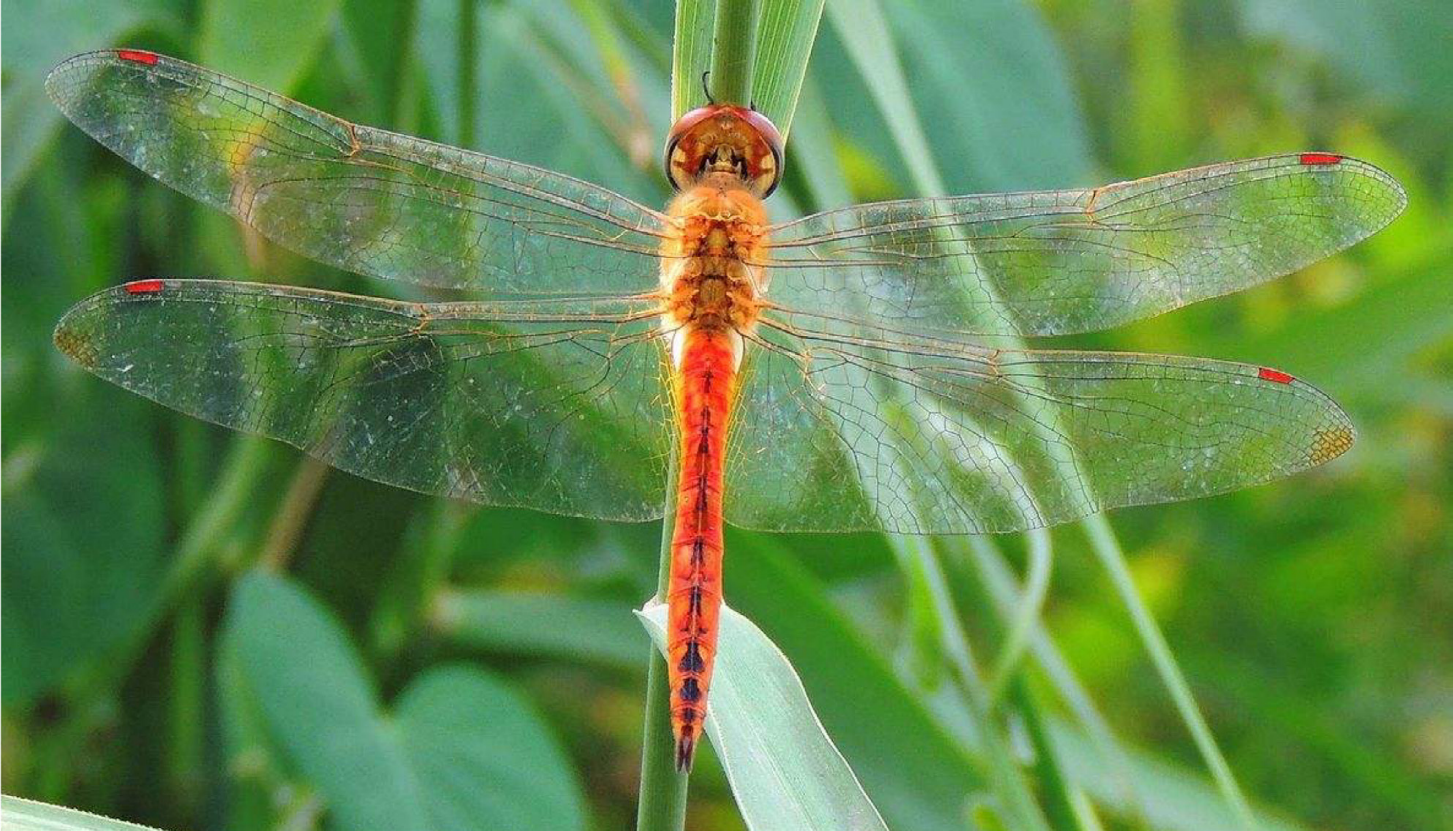
The photo of *P. flavescens*.

The annual and seasonal migration of insects redistributes nutrients, biomass and species, which has a key influence on essential ecosystem functions (Wotton et al., 2019). Meanwhile, mass‐migrating species can interact with other species in places they move through or occupy, which may be important to ecological processes. Every year, billions of dragonflies, butterflies and other insects undertake regular seasonal migrations within and between continents (Wikelski et al., 2006). With a nearly global distribution, *Pantala flavescens* (Fabricius) (Odonata: Libellulidae) (Figure 1) has the longest documented migratory routine in any known insect and undergoes multigenerational transoceanic migration from India to Africa and back again (Hobson et al., 2012). Numerous observations by radar studies have revealed that *P. flavescens* migrate seasonally in northern China, migrating north in summer and south in autumn every year (Cao et al., 2018). Stable isotope tracking of *P. flavescens* has been conducted to elucidate the origins of migrants (Hobson et al., 2021). In recent years, comparative transcriptomic and next‐generation sequencing technologies have been used to explore differences in gene expression between migratory and nonmigratory populations of the species (Talla et al., 2020; Wang et al., 2020; Zhan et al., 2014). For example, some genes related to flight muscle structure, the regulation of hormones and the mobilisation of lipids were revealed in migratory *Helicoverpa armigera* (Jones et al., 2015). Based on differential expression in flight muscles of migratory and non‐migratory monarchs that are correlated with flight metabolic rates, collagen type IV α‐1 has been proposed to regulate flight efficiency during long‐distance migration (Zhan et al., 2014). The two‐way migration of Eastern North American monarchs suggests that epigenetic mechanisms triggered by environmental changes regulate migratory behaviour in this species (Merlin & Liedvogel, 2019). Performing RNA‐seq studies in the brains of fall migrants, fall migrants reprogrammed into spring remigrants by cold treatment and wild‐caught spring remigrants, could be used to detect differentially expressed genes (DEGs) and non‐coding RNAs, as well as cold‐dependent RNA splicing or editing events (Merlin et al., 2020).

In contrast to non‐migratory *P. flavescens*, some genes associated with electron transport chain complexes, microtubules, antioxidants and detoxification were found to be highly expressed in migratory *P. flavescens* (Zhang & Cao, 2023). However, the differences in gene expression profiles in insects between summer and fall migration have rarely been reported.

In this work, our objective was to find a list of the potential DEGs and delineate the function of DEGs between the summer and fall migratory *P. flavescens* by comparing the gene expression profiles. To further ascertain these different genes, we measured the expression of 10 genes in *P. flavescens* by quantitative real‐time PCR analysis.

## 2 MATERIALS AND METHODS

### 2.1 Ethics statement

*Pantala flavescens* is a migratory insect that is widely distributed across the globe. The insect has been evaluated as ‘Least Concern’ species by the International Union for Conservation of Nature (IUCN). In China, *P. flavescens* sampling does not involve endangered or protected species, therefore, no special permits are required for field studies.

### 2.2 Sample collection, RNA extraction and Illumina sequencing

In July and October 2020, migratory *P. flavescens* individuals were trapped using searchlight traps on Beihuang Island (38°23′20″ N, 120°54′52″ E), located in the centre of the Bohai Strait in Changdao County, Shandong Province, China (Fu et al., 2014). There is no fresh water at this site, and no larvae of this species were found during daily observations from spring to fall on Beihuang Island. Fifty samples were rinsed three times in 70% ethanol and then stored at −80°C until transported to the laboratory. The samples were divided into summer migratory individuals trapped in July (M7) and fall migratory individuals trapped in October (M10).

Total RNA was extracted from the thoracic muscle of each sample using TRIzol® Reagent (Invitrogen) according to the manufacturer's instructions. The purity, concentration and integrity of the RNA were verified by a Nanodrop2000 Spectrophotometer (Thermo Fisher Scientific) and 1% agarose gel electrophoresis. Samples with three biological replicates from summer and fall migratory individuals were labelled M7‐1, M7‐2 and M7‐3 and M10‐1, M10‐2 and M10‐3. The RNA used in each biological repetition comes from a mixed RNA of five individuals. In total, six mixed RNA samples were sent to Shanghai Majorbio Bio‐Pharm Technology Co., Ltd. (Shanghai, China) for cDNA library construction and RNA‐seq.

### 2.3 De novo assembly and gene annotation

The raw Illumina sequence data were trimmed and controlled for quality by fastp (https://github.com/OpenGene/fastp) with default parameters (Chen et al., 2018). After filtering, the remaining reads were deemed clean reads and stored in FASTQ format. The clean reads were assembled by using the Trinity assembly program with default parameters (Grabherr et al., 2011). TGICL clustering software v2.1 with default parameters was used to cluster and remove redundancy to obtain nonredundant unigenes (Pertea et al., 2003). The quality of the unigenes was measured using TransRate (v2.4.0). The integrity of the transcriptome assembly was assessed by BUSCO v3.0.2 (Simão et al., 2015).

To annotate the dragonfly transcriptome, we performed a BLAST v2.2.23 search with a cut‐off E‐value of 10^−5^ against six databases: the nonredundant protein sequence (nr) and nucleotide sequence (nt) databases, the KEGG database, the Gene Ontology (GO) database, a manually annotated and reviewed protein sequence database (Swiss‐Prot) and the COG database. The sequence orientations and coding sequences of the unigenes were determined based on the BLASTx best hits from these databases. Gene Ontology annotation of unigenes was conducted by Blast2GO v2.5.0 software through a search of the nr database (Conesa et al., 2005), and WEGO software was used to determine GO functional classification and assess the functional distribution (Ye et al., 2006).

### 2.4 Abundance estimation and identification of DEGs

The unigenes from each sample were compared with the reference unigene database using Bowtie2 v2.1.0 software (Langmead & Salzberg, 2012). The expression level of the unigenes was estimated using RSEM v1.2.12 (Li & Dewey, 2011) with the default parameters for each sample, employing the reads per kilo base per million mapped reads (RPKM) values. DESeq2 was applied for the identification of DEGs in different samples (Love et al., 2014). Through comparison, we used the absolute value of log_2_ ratio ≥ 2 and *p*‐adjust ≤.01 as the threshold to judge the significance level of gene expression differences. GO functional enrichment and KEGG pathway enrichment analyses were carried out by Goatools (https://github.com/tanghaibao/Goatools) and KOBAS (http://kobas.cbi.pku.edu.cn) (Xie et al., 2011). Gene clustering analysis was performed to cluster DEGs with RSEM with *p*‐adjust ≤.05 (Li & Dewey, 2011).

### 2.5 Quantitative real‐time PCR analysis (qRT–PCR validation)

To validate the reliability of the RNA‐Seq data, we randomly selected 10 enriched DEGs for qRT–PCR analysis with *β‐actin* as the reference gene (Ridgeway & Timm, 2015; Zhao et al., 2022). Primers for qRT–PCR are shown in Table 1. The total RNA of each sample was extracted using TRIzol® Reagent (Invitrogen) according to the manufacturer's protocol. HiScript Q RT SuperMix for qPCR (Vazyme, China) was used to synthesise cDNA. qRT–PCR was carried out in a LightCycler® 480 Real‐time PCR system (Roche) using ChamQ Universal SYBR qPCR Master Mix (Vazyme, China). Each reaction was conducted with three independent biological replicates. The relative expression ratio of the target gene was determined using Ct (threshold cycles) and calculated using the 2^−∆∆Ct^ method (Livak & Schmittgen, 2001). The qRT–PCR data were analysed using SPSS v16.0 software.

**TABLE 1:** Primer sequences and unigenes investigated in qPCR.

Unigene ID	Primer sequence
1. TRINITY_DN3519_c0_g1	F: CTCACCACCCTTGTCCTTGC
	R: GGCTGGAATCCGTTCTCGT
2. TRINITY_DN7472_c0_g2	F: TCTTTGCGGTCCTTATCCAT
	R: TCTCCCTAACCTCTGCTCCC
3. TRINITY_DN2499_c0_g1	F: TTCCTCCCACTTCGTCGCT
	R: CGTCGCACAACCTGGTATTT
4. TRINITY_DN6371_c0_g1	F: CTGTTTGCCACCGTTGCTT
	R: GTCCACGATGTCCCGAATG
5. TRINITY_DN1480_c0_g1	F: CAAGAGCCAGGAGGAGGAA
	R: ACGATAGGTTGATGGACGATG
6. TRINITY_DN2295_c0_g2	F: CGAAATCGGGGTGAAGAGG
	R: CCAACCAGCGGTAGAACTATGA
7. TRINITY_DN128_c0_g1	F: AGGCTGGCTATGGAACTGG
	R: CTTTGCGGTAGTGGGGAAT
8. TRINITY_DN8480_c0_g1	F: CTCCAGGGTCCTTATTTTAGTCC
	R: TCAGCCATCCGTTTTCTTTC
9. TRINITY_DN244_c1_g1	F: TTGACGAGAAAGGAAAACCAG
	R: TAACAAAATCAGGAACGGGAG
10. TRINITY_DN4344_c0_g1	F: TAGTAGGAGGCAGCGGTGAG
	R: GGAGATGGTGAAGAGCAAGAAC
β‐Actin	F: TGTGATGGTGGGTATGGGTC
	R: ATGGCTGGGGTATTGAAGG

## 3 RESULTS

### 3.1 Sequencing and de novo assembly

After filtering low‐quality sequences, the clean data for each sample were more than 7.0 Gb, with a total of 316.79 million clean reads, and 46.62 Gb were obtained with 98.3% Q20 and 94.74% Q30 (Table 2). Through the assembly and alignment of the clean reads, 17,810 unigenes and 27,701 transcripts were obtained with an average length of 2046 bp and an N50 value of 3583 bp.

**TABLE 2:** Summary of the transcriptome data filtering.

Sample	Raw reads	Clean reads	Clean bases	Error rate (%)	Q20 (%)	Q30 (%)	GC content (%)
M7_1	48,371,764	48,027,344	7,085,571,260	0.0241	98.4	94.98	35.81
M7_2	53,800,472	53,344,852	7,808,400,995	0.024	98.45	95.11	39.14
M7_3	52,653,226	52,173,194	7,666,226,553	0.0244	98.3	94.74	38.69
M10_1	54,958,422	54,424,308	8,039,591,076	0.024	98.45	95.11	38.85
M10_2	56,009,956	55,547,282	8,188,651,473	0.024	98.45	95.14	40.53
M10_3	53,689,804	53,277,568	7,835,118,127	0.0242	98.38	94.95	38.4

For the 17,810 assembled unigenes, a total of 10,920 unigenes (61.32%) were assigned annotations in at least one database, while the remaining unigenes (38.68%) were of undetermined function. Specifically, 8135 unigenes (45.68%) were matched to known genes in the GO database, 7189 unigenes (40.37%) to genes in the KEGG database, 10,076 unigenes (56.58%) to genes in the eggNOG database, 10,510 unigenes (59.02%) to genes in the NR database, 9050 unigenes (50.82%) to genes in the Swiss‐Prot database and 9496 unigenes (53.32%) to genes in the Pfam database (Table 3).

**TABLE 3:** Summary statistics of annotation of all unigenes.

Databases	Unigene number	Percentage (%)
GO	8135	45.68
KEGG	7189	40.37
eggNOG	10,076	56.58
NR	10,510	59.02
Swiss‐Prot	9050	50.82
Pfam	9496	53.32
Total_annotion	10,920	61.32
Total	17,810	100.00

### 3.2 Gene expression differences between summer and fall migrants

**FIGURE 2:**
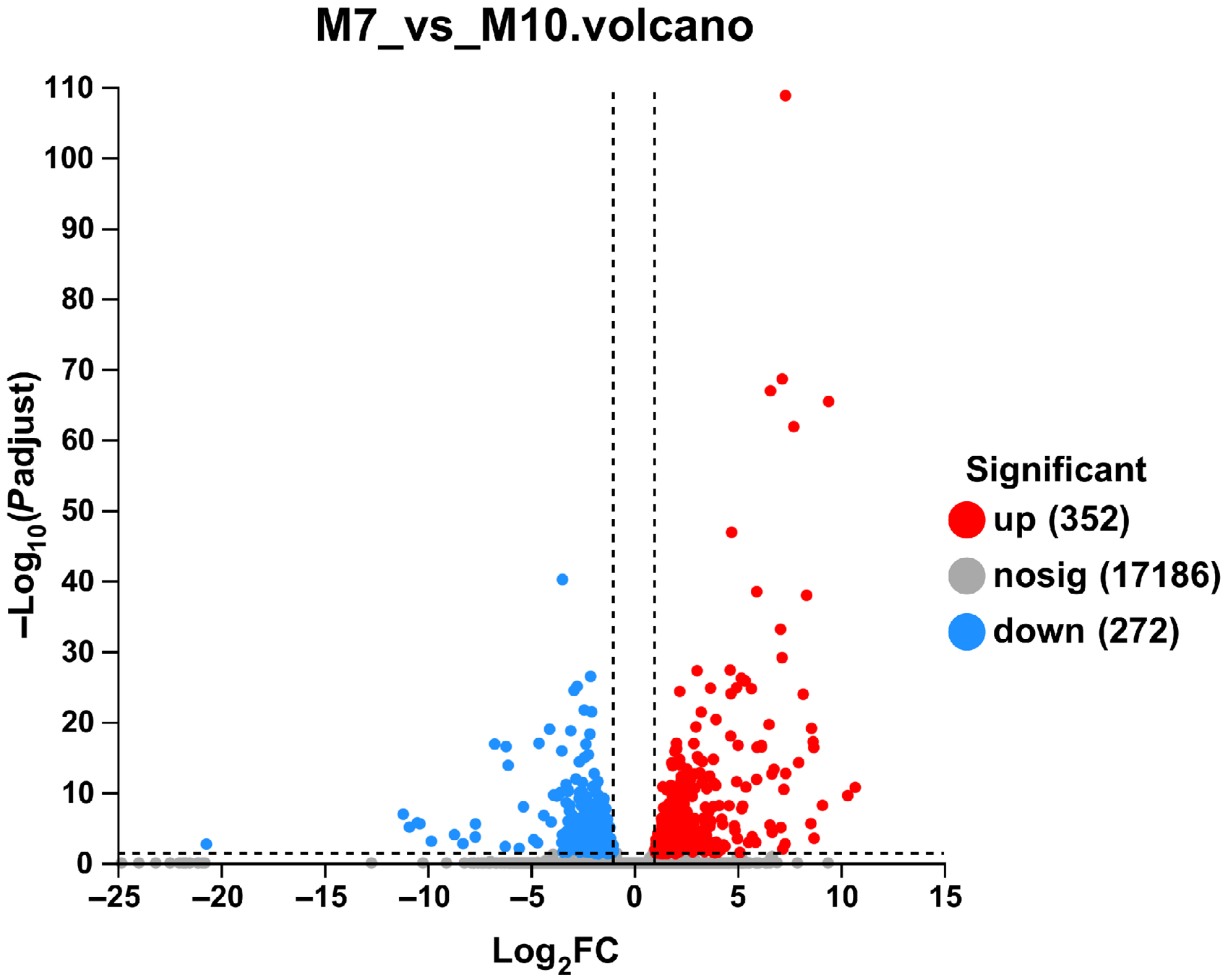
Volcano plots of DEGs in the M7 group versus M10. The x axis represents the log_2_‐fold change, and the y axis represents the negative log_10_ of the corrected *p* value. Blue points denote significantly downregulated genes, and red denotes significantly upregulated genes in each comparison with the criteria of |log_2_(Foldchange)| > 2 and corrected *p* value <.01.

**FIGURE 3:**
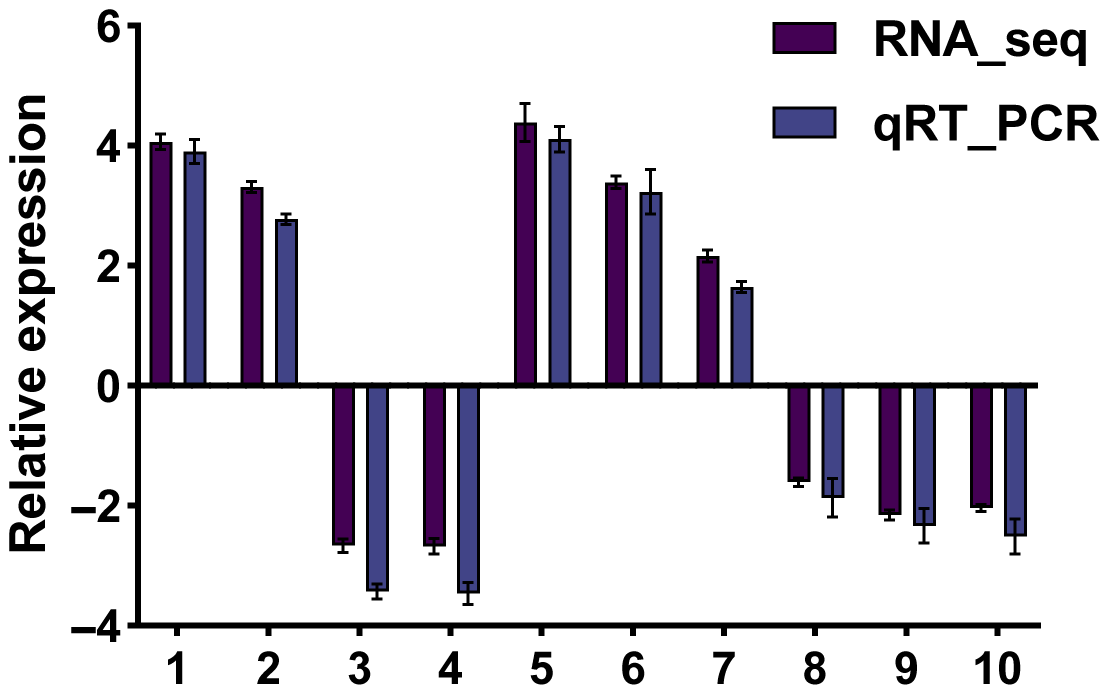
qRT–PCR validation of DEGs. Three replicates were analysed. The *X*‐axis represents different unigenes. The selected genes are listed in Table 1. The *Y*‐axis shows the relative expression levels of genes. *Actin* was used as a reference gene. The error bars indicate standard deviations.

A total of 624 DEGs were identified by DESeq2 in *P. flavescens* between summer and fall migration with the criterion of log_2_ ratio ≥ 2 and a false discovery rate (FDR) of ≤0.01. Among 624 DEGs, there were 352 significantly upregulated and 272 downregulated DEGs in the M7 group compared to the M10 group (Figure 2). The top 15 up‐ and downregulated genes were chosen by the highest expression changes FC (M10/M7) (Table 4). The putative functions of these genes were identified according to the NR database. Among the 15 upregulated genes, the vast majority of upregulated genes were associated with structural constituent of cuticle (11/15). The 15 top downregulated genes were mainly involved in vitellogenin and lipid transporter activity (6/15), defensin and sarcotoxin (3/15) and ion binding (3/15) (Table 4). To validate the RNA‐Seq data, quantitative PCR (qPCR) was used to measure the expression levels of 10 DEGs. The results of the qRT–PCR validation proved that the RNA‐seq data were reliable (Figure 3).

**TABLE 4:** Top 15 upregulated and downregulated genes. List are ordered by FC (M10/M7).

Unigene ID	NR description	FC (M10/M7)	Corrected *p* value	Regulate
TRINIT2 Y_DN2090_c0_g1	Glycine‐rich cell wall structural protein 1.8	1665.035	1.99E−11	Up
TRINITY_DN4075_c0_g1	Structural constituent of cuticle	418.65	.0003188	Up
TRINITY_DN3527_c0_g1	Endocuticle structural glycoprotein SgAbd‐3	414.476	4.44E−17	Up
TRINITY_DN3910_c0_g1	Endocuticle structural glycoprotein ABD‐4	290.784	1.27E−24	Up
TRINITY_DN3577_c0_g1	Cuticle protein‐like	249.855	6.20E−15	Up
TRINITY_DN1537_c0_g1	Integral component of membrane	211.752	1.55E−62	Up
TRINITY_DN5154_c0_g1	Integral component of membrane	161.774	2.14E−13	Up
TRINITY_DN2104_c0_g1	Structural constituent of cuticle	143.748	2.66E−69	Up
TRINITY_DN4975_c0_g1	Structural constituent of cuticle	101.883	2.48E−13	Up
TRINITY_DN2209_c0_g1	Structural constituent of cuticle	97.075	1.28E−67	Up
TRINITY_DN48_c0_g2	Cuticle protein 70, isoforms A and B‐like	71.951	3.74E−17	Up
TRINITY_DN5903_c0_g1	Integral component of membrane	61.809	4.18E−17	Up
TRINITY_DN5490_c0_g1	Structural constituent of cuticle	61.138	3.72E−39	Up
TRINITY_DN4943_c0_g1	Oxidoreductase activity	60.843	1.48E−12	Up
TRINITY_DN11172_c0_g2	Structural constituent of cuticle	51.34	1.99E−04	Up
TRINITY_DN4759_c4_g1	Lipid transporter activity	0	1.25E−07	Down
TRINITY_DN5473_c0_g1	NADH dehydrogenase subunit 5	0	.0021767	Down
TRINITY_DN3379_c0_g1	Vitellogenin	0.001	.0008426	Down
TRINITY_DN4759_c4_g2	Vitellogenin	0.001	2.14E−06	Down
TRINITY_DN4759_c4_g3	Lipid transporter activity	0.001	2.85E−06	Down
TRINITY_DN3688_c0_g1	Haemolymph lipopolysaccharide‐binding protein	0.003	.0018589	Down
TRINITY_DN1873_c0_g1	Defensin‐1	0.005	3.02E−06	Down
TRINITY_DN3566_c0_g1	Metal ion binding	0.005	.0002115	Down
TRINITY_DN439_c0_g1	Protein kinase activity; ATP binding	0.009	1.44E−17	Down
TRINITY_DN3543_c0_g1	Beta‐hexosaminidase subunit alpha isoform X1	0.013	.0047879	Down
TRINITY_DN2313_c0_g1	Sarcotoxin‐2A	0.014	3.24E−17	Down
TRINITY_DN7452_c0_g1	Sarcotoxin‐2A	0.015	1.54E−14	Down
TRINITY_DN2520_c0_g1	Metal ion binding	0.041	1.12E−17	Down
TRINITY_DN8324_c0_g1	Lipid catabolic process	0.049	1.88E−07	Down
TRINITY_DN1077_c0_g1	Zinc ion binding; nucleic acid binding	0.059	1.10E−19	Down

### 3.3 Functional gene enrichment analysis

In vivo, many genes often form networks that coordinate with each other to perform biological functions. Enrichment pathway analysis is helpful to understand the biological function of genes. GO enrichment analysis revealed that 10 GO terms were significantly enriched in the M7 group versus the M10 group, among which the molecular functions (MF) were associated with structural constituent of cuticle, chitin binding, lipid transporter activity and iron‐ion binding; the cell components (CC) were the mitochondria and extracellular region; and the biological processes (BP) were response to bacterium and humoral response (Table 5).

**TABLE 5:** List of the significant results from the gene ontology enrichment analysis and KEGG enrichment analysis between M7 and M10.

GO ID	Term type	Description	Corrected *p* value
GO:0042302	MF	Structural constituent of cuticle	.0027
GO:0008061	MF	Chitin binding	.0027
GO:0009617	BP	Response to bacterium	.0029
GO:0005739	CC	Mitochondrion	.0036
GO:0042742	BP	Defence response to bacterium	.0055
GO:0005319	MF	Lipid transporter activity	.0056
GO:0019731	BP	Antibacterial humoral response	.0073
GO:0019730	BP	Antimicrobial humoral response	.0073
GO:0005506	MF	Iron‐ion binding	.0428
GO:0005576	CC	Extracellular region	.0456

KEGG Pathway id	First category	Description	Corrected *p* value
map05410	Human diseases	Hypertrophic cardiomyopathy	.0132
map00640	Metabolism	Propanoate metabolism	.0171
map00020	Metabolism	Citrate cycle (TCA cycle)	.02248
map05414	Human diseases	Dilated cardiomyopathy	.0278

KEGG is a database resource for the utility of biological systems, such as cells, organisms and ecosystems, and has been constructed from genomic and molecular‐level information. In this study, KEGG enrichment analyses showed that four pathways were significantly enriched, including hypertrophic cardiomyopathy, propanoate metabolism, citrate cycle and dilated cardiomyopathy (Table 5).

### 3.4 Functional gene cluster analysis

**FIGURE 4:**
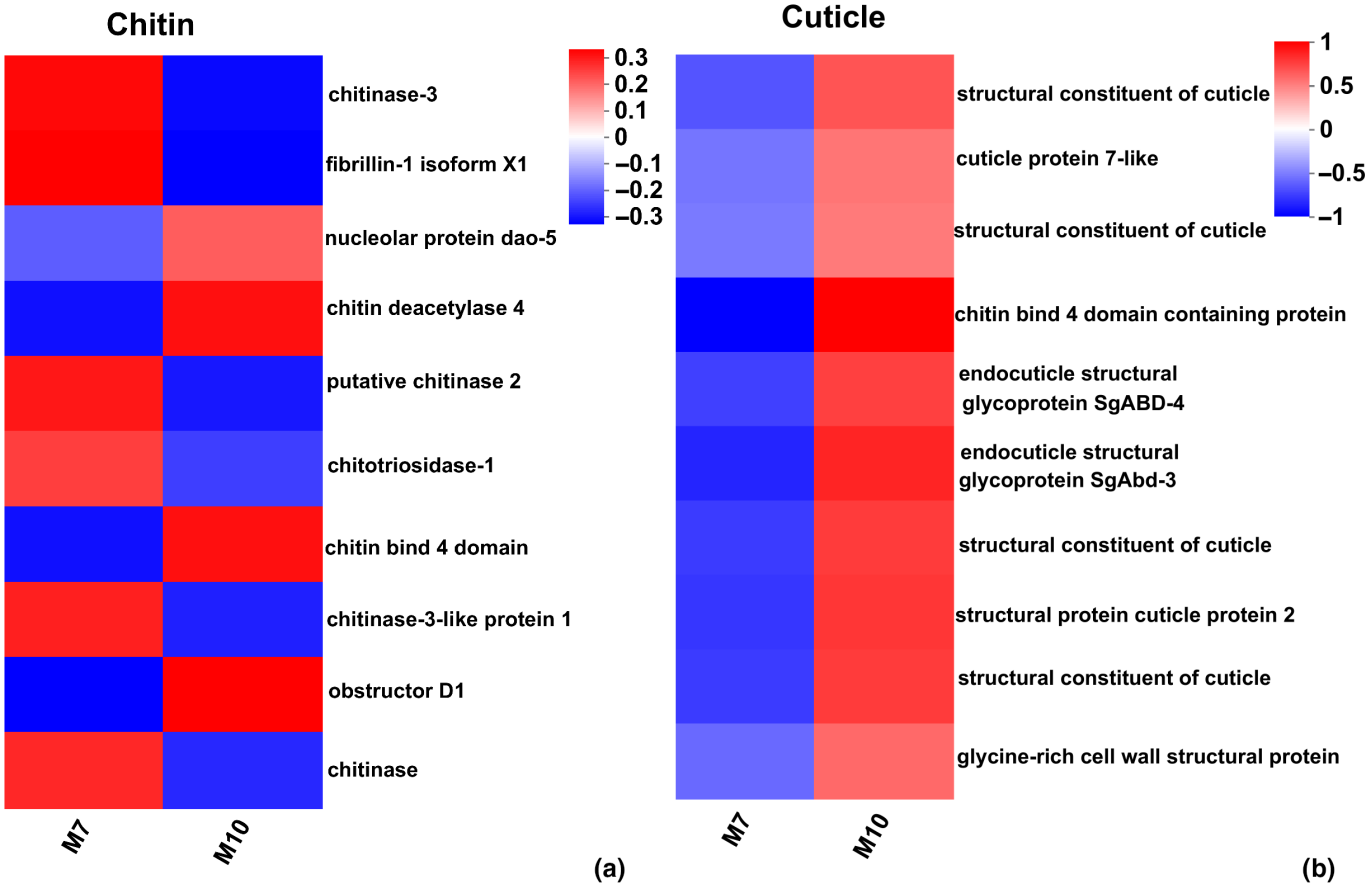
Comparative expression levels of DEGs related to chitin (a) and cuticle (b) in M7 versus M10. The colour scale, which encompasses the lowest (blue) to the highest (red) TPM value, is displayed on the right side.

**FIGURE 5:**
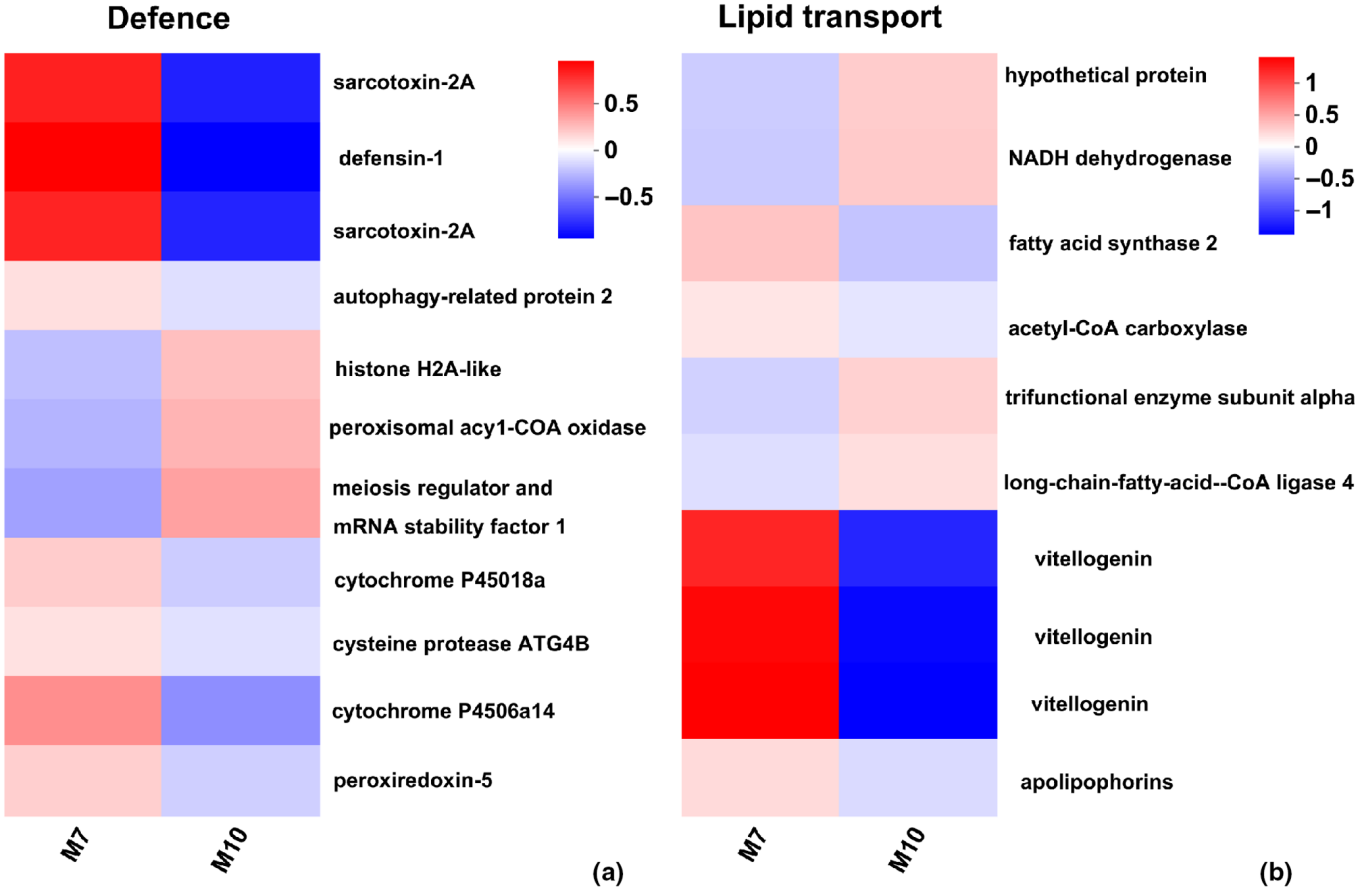
Comparative expression levels of DEGs related to defence (a) and lipid transport (b) in M7 versus M10. The colour scale, which encompasses the lowest (blue) to the highest (red) TPM value, is displayed on the right side.

**FIGURE 6:**
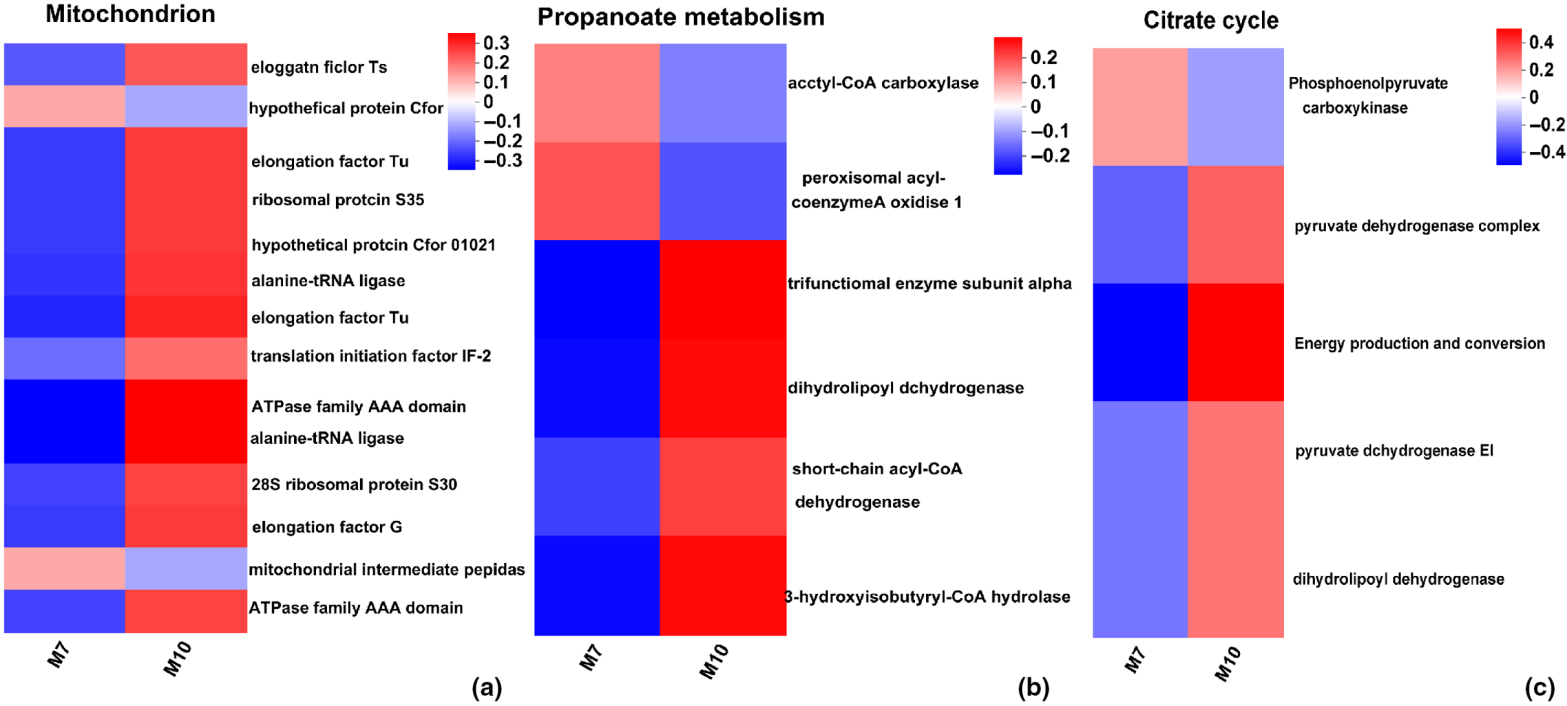
Comparative expression levels of DEGs associated with mitochondrial metabolism (a), propanoate metabolism (b) and citrate cycle (c) in the M7 group versus M10. The colour scale, which encompasses the lowest (blue) to the highest (red) TPM value, is displayed on the right side.

To understand the expression level of different genes in the enrichment pathway, we performed cluster analysis of these genes. The genes involved in the structural constituent of cuticle and chitin were overexpressed, while the expression level of *Chitinase* was downregulated in fall migrants (Figure 4). In the four categories ‘response to bacterium, defence response to bacterium, antibacterial/antimicrobial humoral response’, the genes annotated for sarcotoxin‐2A and defensins were overexpressed in summer migrants (Figure 5a). For the GO term ‘lipid transporter activity’, the expression levels of *vtg2‐*encoded vitellogenin and *fasn2‐*encoded fatty acid synthase 2 were upregulated in summer migrants (Figure 5b). For the GO term ‘mitochondrion’, the genes associated with mitochondrial protein synthesis and energy production were overexpressed in fall migrants (Figure 6a).

**FIGURE 7:**
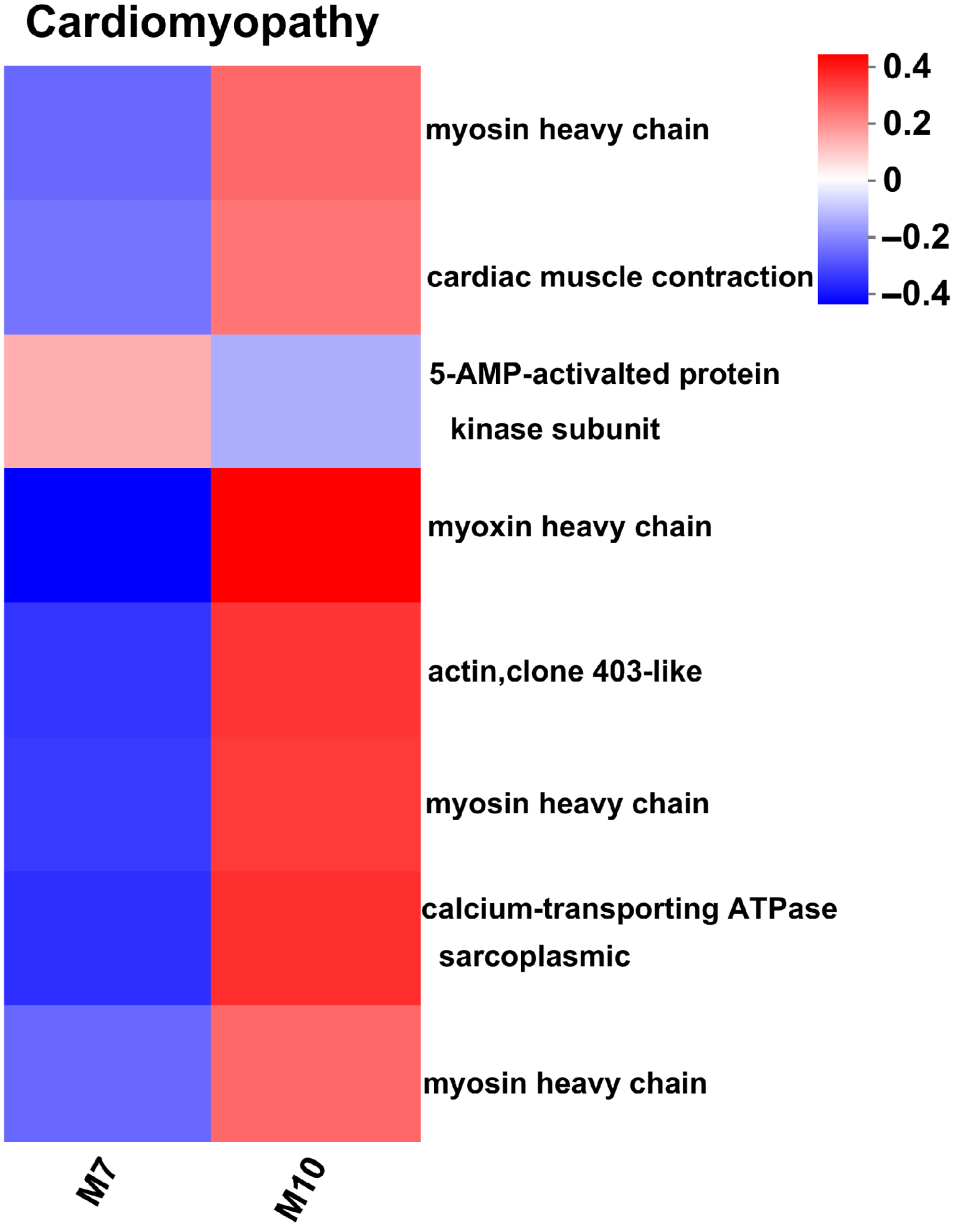
Comparative expression levels of DEGs associated with dilated cardiomyopathy. The colour scale, which encompasses the lowest (blue) to the highest (red) TPM value, is displayed on the right side.

In the propanoate metabolism and citrate cycle enrichment pathways, the expression levels of the genes related to energy production and conversion, such as *mtpbeta‐*encoded trifunctional enzyme subunit alpha, *dld‐1‐*encoded dihydrolipoyl dehydrogenase, *hibch‐*encoded 3‐hydroxyisobutyry1‐COA hydrolase and *pdha‐*encoded pyruvate dehydrogenase, were upregulated in fall migrants (Figure 6b,c). These results indicated that the function of mitochondria was enhanced to release energy when *P. flavescens* were under cold stress. In the hypertrophic cardiomyopathy and dilated cardiomyopathy enrichment pathways, the genes related to movement, such as *mhc‐*encoded myosin heavy chain, *actin*, and *mca‐3* ‐encoded calcium‐transporting ATPase, were upregulated significantly in fall migrants (Figure 7).

## 4 DISCUSSION

The decision to migrate is simultaneously affected by external environmental conditions and the insects' own behaviour and physiological state (Chapman et al., 2015). Seasonal ecosystem linkage was a key ecosystem property for maintaining migratory polymorphism in partially migratory animals (Tanaka et al., 2023). In this study, we identified 624 DEGs, 10 enriched GO pathways and 4 enriched KEEG pathways in the M7 group versus the M10 group. These enrichment pathways are mainly involved in cellular exoskeleton composition, bacterium and humoral response, energy metabolism and muscle contraction. Our findings will help reveal new genes associated with the transoceanic migration of other insects and their impact on global ecology. We have ordered our discussion below based on the DEGs in enriched pathways.

### 4.1 DEGs related to exoskeleton composition

In our study, the expression level of *chitinase* was significantly upregulated in summer migrants (Figure 4a), while the genes annotated for cuticle protein, structural constituent of cuticle and endocuticle structural glycoproteins were significantly upregulated in fall migrants (Figure 4b). Chitin is one of the major components of insect cuticle, which is already known to be a dynamic structure, involved in protecting against water loss, chemical erosion, physical damage and pathogen invasion (Muthukrishnan et al., 2020; Qu et al., 2017). The cuticular structure‐associated transcripts were enriched in many insects exposed to low temperature, suggesting that insect cuticle may play an important role in adaptation to low temperature (Dunning et al., 2014; Huang et al., 2017; Zhou et al., 2019). The individuals in the M10 group had more compact chitin and cuticle layers, protecting the dragonflies against freezing and harm from the external environment. The upregulated expression of *chitinase* can destroy the compact structure of outer epidermis and cuticle of individuals in the M7 group, and make its surface loose to heat dissipation and water evaporation which is suitable for the high temperature in July.

### 4.2 DEGs related to defence and detoxification

Defensins and sarcotoxins are both types of antibacterial proteins and have a large spectrum of antimicrobial action. Defensins are crucial components of the immune response of insects against microbial invasion, parasitic and pathogenic infections (Pasupuleti et al., 2012; Raj & Dentino, 2002). The expression of sarcotoxin II increased strongly in fat bodies after injury and it has been shown to be effective only against a few gram‐negative bacteria (Andoh et al., 2018). The expression of AMP genes including *def1‐*encoded defensin and *loc108905441‐*encoded sarcotoxin II, which protect against bacteria and harmful microorganisms and repair environmental damage, was overexpressed in M7 (Figure 5a), which may be related to the loose binding of the exoskeleton of individuals in this group.

Cytochrome P450 (P450s) are the main detoxifying enzymes involved mainly in the metabolism of a variety of endogenous and exogenous compounds and agrochemical insecticide resistance in insects (Feyereisen, 2012; Jeschke, 2016). For example, overexpression of CYP 6G1 in insects resulted in the increased metabolic detoxification of insecticides (Huang et al., 2013; Joussen et al., 2008). The upregulation of *cyp* expression in M7 group may be related to their higher exposure to toxic substances (Figure 5a).

### 4.3 DEGs related to energy consumption

Vitellogenin encodes the major yolk protein precursor and plays an essential role in the reproduction and spread of all oviparous species, including insects (Tufail et al., 2010). Vitellogenin is not only important for bee reproduction but also involved in their immune response (Harwood et al., 2019; Sun & Zhang, 2015), antioxidative stress response (Seehuus et al., 2006; Salmela et al., 2016) and metal ion transport (Amdam et al., 2004). In the lipid transport term, the expression level of *vtg2* was greatly upregulated in the M7 group, which may have been related to storage of energy, antioxidant activity and metal ion transport in the individuals of this group (Figure 5b). The expression levels of *fasn2‐* and *apoe‐*encoded apolipoprotein were upregulated in the M7 group, which may indicate that individuals stored more fat in this group (Figure 5b).

Mitochondria are essential organelles that execute and coordinate various metabolic pathways involved in ATP synthesis through the tricarboxylic acid cycle and oxidative phosphorylation in the cell (Ng et al., 2021). The genes involved in ribosome assembly and protein synthesis were overexpressed in M10, which indicates the synthesis of a great number of proteins and ATP production in the mitochondria (Figure 6a). Trifunctional enzyme subunits α and β compose mitochondrial trifunctional proteins that catalyse the last three steps in long‐chain fatty acid metabolism for energy supply (van Vliet et al., 2018). The expression levels of *mtpbeta* and *acads* encoded short‐chain acyl‐CoA dehydrogenase upregulated in the M10 group can promote fatty acid metabolism (Figure 6b). The pyruvate dehydrogenase complex composed of pyruvate dehydrogenase, dihydrolipoyl transacetylase and dihydrolipoyl dehydrogenase catalyses, the oxidative decarboxylation of pyruvate to acetyl coenzyme A, linking glycolysis to the tricarboxylic acid cycle (Holness & Sugden, 2003). The expression levels of *pdha and dld‐1* were significantly upregulated in the M10 group, which can accelerate glycolysis, the tricarboxylic acid cycle and ATP production (Figure 6c).

### 4.4 DEGs related to muscle contraction

Sarcomeric myosin heavy‐chain (MYH) genes are expressed in the major cardiac and skeletal muscles. Myosin produces the force necessary for a variety of cellular movements by hydrolysing ATP and interacting with actin filaments (Lee et al., 2019). The high expression levels of *mhc* and *actin* indicated that contractions and cellular movements of individuals in the M10 group were more frequent and intense (Figure 7). The cardiac isoform of the sarcoplasmic/endoplasmic reticulum calcium ATPase (SERCA2a) is activated by ATP hydrolysis. SERCA2a downregulation leads to a deficient contractile state, while SERCA2a overexpression has beneficial effects in a host of other cardiovascular diseases (Kósa et al., 2015). The overexpression of *serca* in the M10 group may also be good for muscle contraction (Figure 7).

## 5 CONCLUSION

We compared the transcriptomes of *P. flavescens* under different seasons using RNA‐Seq technology based on high‐throughput sequencing. A large number of DEGs were discovered in metabolic pathways through GO and KEGG enrichment analysis. Our data showed migratory dragonflies in fall might have stronger flight intensity, more energy expenditure and tighter chitin bonding than those in summer. Migratory dragonflies in summer might have more resistance and detoxification substances. These changes in gene expression may be more conducive to their survival. These newly found genes may provide a molecular basis for evaluating the migratory flight of insects and identifying key genes involved in different life‐history strategies. However, our results cannot represent gene expression differences in other tissues, such as the head, brain and ovary. The regulatory mechanisms of insect migratory behaviour and physiological changes are still poorly understood and need to be further explored from multiple perspectives.

## AUTHOR CONTRIBUTIONS

**Lingzhen Cao:** Conceptualization (equal); funding acquisition (equal); writing – original draft (equal); writing – review and editing (equal). **Na Wang:** Investigation (equal); methodology (equal); validation (equal); writing – original draft (equal).

## CONFLICT OF INTEREST STATEMENT

The authors declare no conflicts of interest.

## Data Availability

The data that support the findings of this study are openly available in the Short Archive (SRA) of NCBI at https://www.ncbi.nlm.nih.gov/sra/?term=PRJNA762591 (reference number: PRJNA 762591).

## References

[ece370147-bib-0001] Amdam, G. V. , Simões, Z. L. , Hagen, A. , Norberg, K. , Schrøder, K. , Mikkelsen, Ø. , Kirkwood, T. B. L. , & Omholt, S. W. (2004). Hormonal control of the yolk precursor vitellogenin regulates immune function and longevity in honeybees. Experimental Gerontology, 39(5), 767–773. 15130671 10.1016/j.exger.2004.02.010

[ece370147-bib-0002] Andoh, M. , Ueno, T. , & Kawasaki, K. (2018). Tissue‐dependent induction of antimicrobial peptide genes after body wall injury in house fly (Musca domestica) larvae. Drug Discoveries & Therapeutics, 12(6), 355–362. 30674770 10.5582/ddt.2018.01063

[ece370147-bib-0003] Cao, L. Z. , Fu, X. W. , Hu, C. X. , & Wu, K. M. (2018). Seasonal migration of *Pantala flavescens* across the Bohai Strait in northern China. Environmental Entomology, 47(2), 264–270. 29546318 10.1093/ee/nvy017

[ece370147-bib-0004] Chapman, J. W. , Reynolds, D. R. , & Wilson, K. (2015). Long‐range seasonal migration in insects: Mechanisms, evolutionary drivers and ecological consequences. Ecology Letters, 18(3), 287–302. 25611117 10.1111/ele.12407

[ece370147-bib-0005] Chen, S. , Zhou, Y. , Chen, Y. , & Gu, J. (2018). fastp: An ultra‐fast all‐in‐one FASTQ preprocessor. Bioinformatics, 34(17), i884–i890. 30423086 10.1093/bioinformatics/bty560 PMC6129281

[ece370147-bib-0006] Conesa, A. , Götz, S. , García‐Gómez, J. M. , Terol, J. , Talón, M. , & Robles, M. (2005). Blast2GO: A universal tool for annotation, visualization and analysis in functional genomics research. Bioinformatics, 21(18), 3674–3676. 16081474 10.1093/bioinformatics/bti610

[ece370147-bib-0007] Cui, J. , Zhu, S. Y. , Bi, R. , Xu, W. , Gao, Y. , & Shi, S. S. (2018). Effect of temperature on the development, survival, and fecundity of *Heliothis viriplaca* (Lepidoptera: Noctuidae). Journal of Economic Entomology, 111(4), 1940–1946. 29905847 10.1093/jee/toy151 PMC6074835

[ece370147-bib-0008] Dunning, L. T. , Dennis, A. B. , Sinclair, B. J. , Newcomb, R. D. , & Buckley, T. R. (2014). Divergent transcriptional responses to low temperature among populations of alpine and lowland species of New Zealand stick insects (*Micrarchus*). Molecular Ecology, 23(11), 2712–2726. 24762129 10.1111/mec.12767

[ece370147-bib-0009] Feyereisen, R. (2012). Insect CYP genes and P450 enzymes. Insect Molecular Biology and Biochemistry, 8, 236–316.

[ece370147-bib-0010] Fu, X. W. , Xing, Z. L. , Liu, Z. F. , Ali, A. , & Wu, K. M. (2014). Migration of diamondback moth, *Plutella xylostella*, across the Bohai Sea in northern China. Crop Protection, 64, 143–149.

[ece370147-bib-0011] Grabherr, M. G. , Haas, B. J. , Yassour, M. , Levin, J. Z. , Thompson, D. A. , Amit, I. , Adiconis, X. , Fan, L. , Raychowdhury, R. , Zeng, Q. , Chen, Z. , Mauceli, E. , Hacohen, N. , Gnirke, A. , Rhind, N. , di Palma, F. , Birren, B. W. , Nusbaum, C. , Lindblad‐Toh, K. , … Regev, A. (2011). Full‐length transcriptome assembly from RNA‐Seq data without a reference genome. Nature Biotechnology, 29(7), 644–652. 10.1038/nbt.1883 PMC3571712 21572440

[ece370147-bib-0012] Harwood, G. , Amdam, G. , & Freitak, D. (2019). The role of Vitellogenin in the transfer of immune elicitors from gut to hypopharyngeal glands in honey bees (*Apis mellifera*). Journal of Insect Physiology, 112, 90–100. 30578811 10.1016/j.jinsphys.2018.12.006

[ece370147-bib-0013] Hobson, K. A. , Anderson, R. C. , Soto, D. X. , & Wassenaar, L. I. (2012). Isotopic evidence that dragonflies (*Pantala flavescens*) migrating through the Maldives come from the northern Indian subcontinent. PLoS One, 7(12), e52594. 23285106 10.1371/journal.pone.0052594 PMC3527571

[ece370147-bib-0014] Hobson, K. A. , Jinguji, H. , Ichikawa, Y. , Kusack, J. W. , & Anderson, R. C. (2021). Long‐distance migration of the globe skimmer dragonfly to Japan revealed using stable hydrogen (delta H‐2) isotopes. Environmental Entomology, 50(1), 247–255. 33219373 10.1093/ee/nvaa147

[ece370147-bib-0015] Holness, M. J. , & Sugden, M. C. (2003). Regulation of pyruvate dehydrogenase complex activity by reversible phosphorylation. Biochemical Society Transactions, 31(6), 1143–1151. 14641014 10.1042/bst0311143

[ece370147-bib-0016] Huang, H. J. , Xue, J. , Zhuo, J. C. , Cheng, R. L. , Xu, H. J. , & Zhang, C. X. (2017). Comparative analysis of the transcriptional responses to low and high temperatures in three rice planthopper species. Molecular Ecology, 26(10), 2726–2737. 28214356 10.1111/mec.14067

[ece370147-bib-0017] Huang, Y. , Shen, G. M. , Jiang, H. B. , Jiang, X. Z. , Dou, W. , & Wang, J. J. (2013). Multiple P450 genes: Identification, tissue‐specific expression and their responses to insecticide treatments in the oriental fruit fly, *Bactrocera dorsalis* (Hendel) (Diptera: Tephritidea). Pesticide Biochemistry and Physiology, 106(1–2), 1–7.

[ece370147-bib-0018] Jeschke, P. (2016). Propesticides and their use as agrochemicals. Pest Management Science, 72(2), 210–225. 26449612 10.1002/ps.4170

[ece370147-bib-0019] Jones, C. M. , Papanicolaou, A. , Mironidis, G. K. , Vontas, J. , & Chapman, J. W. (2015). Genomewide transcriptional signatures of migratory flight activity in a globally invasive insect pest. Molecular Ecology, 24(19), 4901–4911. 26331997 10.1111/mec.13362 PMC5102652

[ece370147-bib-0020] Joussen, N. , Heckel, D. G. , Haas, M. , Schuphan, I. , & Schmidt, B. (2008). Metabolism of imidacloprid and DDT by P450 CYP6G1 expressed in cell cultures of *Nicotiana tabacum* suggests detoxification of these insecticides in Cyp6g1‐overexpressing strains of *Drosophila melanogaster* leading to resistance. Pest Management Science, 64(1), 65–73. 17912692 10.1002/ps.1472

[ece370147-bib-0021] Kósa, M. , Brinyiczki, K. , van Damme, P. , Goemans, N. , Hancsák, K. , Mendler, L. , & Zádor, E. (2015). The neonatal sarcoplasmic reticulum Ca2+‐ATPase gives a clue to development and pathology in human muscles. Journal of Muscle Research and Cell Motility, 36(2), 195–203. 25487304 10.1007/s10974-014-9403-z

[ece370147-bib-0022] Langmead, B. , & Salzberg, S. L. (2012). Fast gapped‐read alignment with bowtie 2. Nature Methods, 9(4), 357–359. 22388286 10.1038/nmeth.1923 PMC3322381

[ece370147-bib-0023] Lee, L. A. , Karabina, A. , Broadwell, L. J. , & Leinwand, L. A. (2019). The ancient sarcomeric myosins found in specialized muscles. Skeletal Muscle, 9(1), 7. 30836986 10.1186/s13395-019-0192-3 PMC6402096

[ece370147-bib-0024] Li, B. , & Dewey, C. N. (2011). RSEM: Accurate transcript quantification from RNA‐Seq data with or without a reference genome. BMC Bioinformatics, 12, 323. 21816040 10.1186/1471-2105-12-323 PMC3163565

[ece370147-bib-0025] Livak, K. J. , & Schmittgen, T. D. (2001). Analysis of relative gene expression data using real‐time quantitative PCR and the 2(‐Delta Delta C(T)) method. Methods (San Diego, Calif.), 25(4), 402–408. 11846609 10.1006/meth.2001.1262

[ece370147-bib-0026] Love, M. I. , Huber, W. , & Anders, S. (2014). Moderated estimation of fold change and dispersion for RNA‐seq data with DESeq2. Genome Biology, 15(12), 550. 25516281 10.1186/s13059-014-0550-8 PMC4302049

[ece370147-bib-0027] Merlin, C. , Iiams, S. E. , & Lugena, A. B. (2020). Monarch butterfly migration moving into the genetic era. Trends in Genetics, 36(9), 689–701. 32713598 10.1016/j.tig.2020.06.011 PMC9298955

[ece370147-bib-0028] Merlin, C. , & Liedvogel, M. (2019). The genetics and epigenetics of animal migration and orientation: Birds, butterflies and beyond. The Journal of Experimental Biology, 222(Suppl_1), jeb191890. 30728238 10.1242/jeb.191890

[ece370147-bib-0029] Muthukrishnan, S. , Mun, S. , Noh, M. Y. , Geisbrecht, E. R. , & Arakane, Y. (2020). Insect cuticular chitin contributes to form and function. Current Pharmaceutical Design, 26(29), 3530–3545. 32445445 10.2174/1381612826666200523175409 PMC7755156

[ece370147-bib-0030] Ng, M. Y. W. , Wai, T. , & Simonsen, A. (2021). Quality control of the mitochondrion. Developmental Cell, 56(7), 881–905. 33662258 10.1016/j.devcel.2021.02.009

[ece370147-bib-0031] Pasupuleti, M. , Schmidtchen, A. , & Malmsten, M. (2012). Antimicrobial peptides: Key components of the innate immune system. Critical Reviews in Biotechnology, 32(2), 143–171. 22074402 10.3109/07388551.2011.594423

[ece370147-bib-0032] Pertea, G. , Huang, X. , Liang, F. , Antonescu, V. , Sultana, R. , Karamycheva, S. , Lee, Y. , White, J. , Cheung, F. , Parvizi, B. , Tsai, J. , & Quackenbush, J. (2003). TIGR gene indices clustering tools (TGICL): A software system for fast clustering of large EST datasets. Bioinformatics (Oxford, England), 19(5), 651–652. 12651724 10.1093/bioinformatics/btg034

[ece370147-bib-0033] Qu, M. , Ren, Y. , Liu, Y. , & Yang, Q. (2017). Studies on the chitin/chitosan binding properties of six cuticular proteins analogous to peritrophin 3 from Bombyx mori. Insect Molecular Biology, 26(4), 432–439. 28432772 10.1111/imb.12308

[ece370147-bib-0034] Raj, P. A. , & Dentino, A. R. (2002). Current status of defensins and their role in innate and adaptive immunity. FEMS Microbiology Letters, 206(1), 9–18. 11786250 10.1111/j.1574-6968.2002.tb10979.x

[ece370147-bib-0035] Ridgeway, J. A. , & Timm, A. E. (2015). Reference gene selection for quantitative real‐time PCR normalization in larvae of three species of Grapholitini (Lepidoptera: Tortricidae). PLoS One, 10(6), e0129026. 26030743 10.1371/journal.pone.0129026 PMC4450875

[ece370147-bib-0036] Salmela, H. , Stark, T. , Stucki, D. , Fuchs, S. , Freitak, D. , Dey, A. , Kent, C. F. , Zayed, A. , Dhaygude, K. , Hokkanen, H. , & Sundström, L. (2016). Ancient duplications have led to functional divergence of Vitellogenin‐like genes potentially involved in inflammation and oxidative stress in honey bees. Genome Biology and Evolution, 8(3), 495–506. 26961250 10.1093/gbe/evw014 PMC4825421

[ece370147-bib-0037] Satterfield, D. A. , Sillett, T. S. , Chapman, J. W. , Altizer, S. , & Marra, P. P. (2020). Seasonal insect migrations: Massive, influential and overlooked. Frontiers in Ecology and the Environment, 18(6), 335–344.

[ece370147-bib-0038] Seehuus, S. C. , Norberg, K. , Gimsa, U. , Krekling, T. , & Amdam, G. V. (2006). Reproductive protein protects functionally sterile honey bee workers from oxidative stress. Proceedings of the National Academy of Sciences of the United States of America, 103(4), 962–967. 16418279 10.1073/pnas.0502681103 PMC1347965

[ece370147-bib-0039] Simão, F. A. , Waterhouse, R. M. , Ioannidis, P. , Kriventseva, E. V. , & Zdobnov, E. M. (2015). BUSCO: Assessing genome assembly and annotation completeness with single‐copy orthologs. Bioinformatics, 31(19), 3210–3212. 26059717 10.1093/bioinformatics/btv351

[ece370147-bib-0040] Sun, C. , & Zhang, S. (2015). Immune‐relevant and antioxidant activities of Vitellogenin and yolk proteins in fish. Nutrients, 7(10), 8818–8829. 26506386 10.3390/nu7105432 PMC4632452

[ece370147-bib-0041] Talla, V. , Pierce, A. A. , Adams, K. L. , de Man, T. J. B. , Nallu, S. , Villablanca, F. X. , Kronforst, M. R. , & de Roode, J. C. (2020). Genomic evidence for gene flow between monarchs with divergent migratory phenotypes and flight performance. Molecular Ecology, 29(14), 2567–2582. 32542770 10.1111/mec.15508 PMC8118118

[ece370147-bib-0042] Tanaka, T. , Ueda, R. , & Sato, T. (2023). Seasonal ecosystem linkages contribute to the maintenance of migratory polymorphism in a salmonid population. Proceedings. Biological sciences, 290(1995), 20230126. 36946118 10.1098/rspb.2023.0126 PMC10031421

[ece370147-bib-0043] Tufail, M. , Naeemullah, M. , Elmogy, M. , Sharma, P. N. , Takeda, M. , & Nakamura, C. (2010). Molecular cloning, transcriptional regulation, and differential expression profiling of vitellogenin in two wing‐morphs of the brown planthopper, *Nilaparvata lugens Stål* (Hemiptera: Delphacidae). Insect Molecular Biology, 19(6), 787–798. 20698901 10.1111/j.1365-2583.2010.01035.x

[ece370147-bib-0044] van Vliet, P. , Berden, A. E. , van Schie, M. K. M. , Bakker, J. A. , Heringhaus, C. , de Coo, I. F. M. , Langeveld, M. , Schroijen, M. A. , & Arbous, M. S. (2018). Peripheral neuropathy, episodic rhabdomyolysis, and hypoparathyroidism in a patient with mitochondrial trifunctional protein deficiency. JIMD Reports, 38, 101–105. 28685493 10.1007/8904_2017_37 PMC5874207

[ece370147-bib-0045] Wang, S. , Minter, M. , Homem, R. A. , Michaelson, L. V. , Venthur, H. , Lim, K. S. , Withers, A. , Xi, J. , Jones, C. M. , & Zhou, J. (2020). Odorant binding proteins promote flight activity in the migratory insect, *Helicoverpa armigera* . Molecular Ecology, 29(19), 3795–3808. 32681685 10.1111/mec.15556

[ece370147-bib-0046] Wikelski, M. , Moskowitz, D. , Adelman, J. S. , Cochran, J. , Wilcove, D. S. , & May, M. L. (2006). Simple rules guide dragonfly migration. Biology Letters, 2(3), 325–329. 17148394 10.1098/rsbl.2006.0487 PMC1686212

[ece370147-bib-0047] Wotton, K. R. , Gao, B. , Menz, M. H. M. , Morris, R. K. A. , Ball, S. G. , Lim, K. S. , Reynolds, D. R. , Hu, G. , & Chapman, J. W. (2019). Mass seasonal migrations of Hoverflies provide extensive pollination and crop protection services. Current Biology, 29(13), 2167–2173.e5. 31204159 10.1016/j.cub.2019.05.036

[ece370147-bib-0048] Xie, C. , Mao, X. , Huang, J. J. , Ding, Y. , Wu, J. M. , Dong, S. , Kong, L. , Gao, G. , Li, Y. , & Wei, L. P. (2011). KOBAS 2.0: A web server for annotation and identification of enriched pathways and diseases. Nucleic Acids Research, 39(suppl_2), W316–W322. 21715386 10.1093/nar/gkr483 PMC3125809

[ece370147-bib-0049] Ye, J. , Fang, L. , Zheng, H. , Zhang, Y. , Chen, J. , Zhang, Z. , Wang, J. , Li, S. , Li, R. , Bolund, L. , & Wang, J. (2006). WEGO: A web tool for plotting GO annotations. Nucleic Acids Research, 34(suppl_2), W293–W297. 16845012 10.1093/nar/gkl031 PMC1538768

[ece370147-bib-0050] Zhan, S. , Zhang, W. , Niitepõld, K. , Hsu, J. , Haeger, J. F. , Zalucki, M. P. , Altizer, S. , de Roode, J. C. , Reppert, S. M. , & Kronforst, M. R. (2014). The genetics of monarch butterfly migration and warning colouration. Nature, 514, 317–321. 25274300 10.1038/nature13812 PMC4331202

[ece370147-bib-0051] Zhang, D. , & Cao, L. Z. (2023). Transcriptome analysis reveals differentially expressed genes in migratory and nonmigratory *Pantala flavescens* . Annales Zoologici Fennici, 60(1), 73–83.

[ece370147-bib-0052] Zhao, X. , Geng, Y. , Hu, T. , Zhao, Y. , Yang, S. , & Hao, D. (2022). Evaluation of optimal reference genes for qRT‐PCR analysis in *Hyphantria cunea* (Drury). Insects, 13(1), 97. 35055939 10.3390/insects13010097 PMC8778541

[ece370147-bib-0053] Zhou, X. R. , Shan, Y. M. , Tan, Y. , Zhang, Z. R. , & Pang, B. P. (2019). Comparative analysis of transcriptome responses to cold stress in *Galeruca daurica* (Coleoptera: Chrysomelidae). Journal of Insect Science, 19(6), 8. 10.1093/jisesa/iez109 PMC6871913 31752020

